# Complete genome analysis demonstrates multiple introductions of enterovirus 71 and coxsackievirus A16 recombinant strains into Thailand during the past decade

**DOI:** 10.1038/s41426-018-0215-x

**Published:** 2018-12-14

**Authors:** Pirom Noisumdaeng, Kantima Sangsiriwut, Jarunee Prasertsopon, Chompunuch Klinmalai, Sunchai Payungporn, Anek Mungaomklang, Kulkanya Chokephaibulkit, Rome Buathong, Arunee Thitithanyanont, Pilaipan Puthavathana

**Affiliations:** 1 Faculty of Public Health, Thammasat University (Rangsit center), Khlong Luang, Pathum Thani 12121 Thailand; 2 Department of Preventive and Social Medicine, Faculty of Medicine Siriraj Hospital, Mahidol University, Bangkok-noi, Bangkok, 10700 Thailand; 3 Center for Research and Innovation, Faculty of Medical Technology, Mahidol University, Nakhon, Pathom 73170 Thailand; 4 Department of Pediatrics, Faculty of Medicine, Ramathibodi Hospital, Mahidol University, Bangkok, 10400 Thailand; 5 Department of Biochemistry, Faculty of Medicine, Chulalongkorn University, Bangkok, 10330 Thailand; 6 Debaratana Nakhon Ratchasima Hospital, Ministry of Public Health, Nakhon Ratchasima, 30280 Thailand; 7 Department of Pediatrics, Faculty of Medicine Siriraj Hospital, Mahidol University, Bangkok-noi, Bangkok, 10700 Thailand; 8 Bureau of Epidemiology, Department of Disease Control, Ministry of Public Health, Nonthaburi, 11000 Thailand; 9 Department of Microbiology, Faculty of Science, Mahidol University, Bangkok, 10400 Thailand; 10 Department of Microbiology, Faculty of Medicine Siriraj Hospital, Mahidol University, Bangkok-noi, Bangkok, 10700 Thailand

## Abstract

Hand, foot, and mouth disease (HFMD) caused by enteroviruses remains a public health threat, particularly in the Asia-Pacific region during the past two decades. Moreover, the introduction of multiple subgenotypes and the emergence of recombinant viruses is of epidemiological importance. Based on either the full genome or VP1 sequences, 32 enteroviruses (30 from HFMD patients, 1 from an encephalitic patient, and 1 from an asymptomatic contact case) isolated in Thailand between 2006 and 2014 were identified as 25 enterovirus 71 (EV71) isolates (comprising 20 B5, 1 C2, 2 C4a, and 2 C4b subgenotypes) and 7 coxsackievirus A16 (CA16) isolates (comprising 6 B1a and 1 B1b subgenotypes). The EV71 subgenotype C4b was introduced into Thailand for the first time in 2006 and was replaced by subgenotype C4a strains in 2009. Phylogenetic, similarity plot and bootscan analyses of the complete viral genomes identified 12 recombinant viruses among the 32 viral isolates. Only one EV71-B5 isolate out of 20 was a recombinant virus with one region of intratypic or intertypic recombination, while all four EV71-C4 isolates were recombinant viruses having undergone double recombination, and all seven CA16 isolates were recombinant viruses. The recombination breakpoints of these recombinants are located solely within the P2 and P3 regions. Surveillance for circulating strains and subgenotype replacement are important with respect to molecular epidemiology and the selection of the upcoming EV71 vaccine. In addition, the clinical importance of recombinant viruses needs to be further explored.

## Introduction

Hand, foot, and mouth disease (HFMD) is an acute febrile illness characterized by vesicular eruptions on the palms of the hands, the feet, and in the oral cavity and is common in children under 5 years of age worldwide^1–4^. HFMD was recognized as an emerging disease after large outbreaks of the disease in Malaysia in 1997 and Taiwan in 1998^5–8^. Subsequently, outbreaks of HFMD were reported in several part of the Asia-Pacific region. Currently, HFMD is an endemic disease in the People’s Republic of China, Taiwan, and several Southeast Asian countries, such as Vietnam and Cambodia^9–15^. In Thailand, HFMD has been listed as a disease to be notified to the Ministry of Public Health since 2001.

HFMD is caused by several enteroviruses, with the major etiologic agents being enterovirus 71 (EV71) and coxsackievirus A16 (CA16)^3,16^. Compared with CA16, EV71 is associated with relatively more severe symptoms and complications that may involve central nervous system and cardiopulmonary complications, such as brain stem encephalitis, autonomous nervous system dysregulation, neurological sequelae, neurogenic pulmonary edema, and myocarditis^1,4,17,18^. According to epidemiological data, neurological complications in EV71-associated HFMD have varied among outbreaks, suggesting that the viral genetic background has an effect on the disease presentation^11,15^.

EV71 and CA16 belong to the species *Enterovirus A* (EV-A), genus *Enterovirus*, family *Picornaviridae*. Members of this species have small, nonenveloped viral particles with icosahedral symmetry containing a positive sense, single-stranded RNA genome that is ~7.4 kilobases (kb) in length^19,20^. The genome is flanked by a 5ʹ-untranslated region (5ʹ-UTR) adjacent to the virus-encoded peptide (VPg) and a 3ʹ-untranslated region (3ʹ-UTR) adjacent to a poly(A) tail. The genome comprises one open reading frame (ORF) encoding a polyprotein that is divided into three parts, P1, P2, and P3. The P1 region encodes four structural viral proteins (VP1–VP4), while P2 encodes three nonstructural proteins (2A–2C) and P3 encodes for four nonstructural proteins (3A–3D)^3,4,21^. VP1, the major antigenic and neutralizing domain, is the most variable protein. Based on VP1, EV71 is classified into three genotypes, namely, A, B, and C. Genotype A contains a single member, the BrCr prototype, which was initially identified in California, USA, in 1970 from a patient with encephalitis^21,22^. Genotypes B and C are further subdivided into five subgenotypes, including B1-B5 and C1-C5, respectively^4,21^. Similarly, based on VP1 sequences, CA16 is divided into two lineages (A and B). The prototype G-10 is the only member of lineage A, while lineage B is further divided into sublineages B1 (B1a, B1b, and B1c) and B2^23^. In another classification system based on VP4 sequences, CA16 strains isolated between 1999 and 2004 were classified into three lineages, A, B, and C^24^.

Mutation and recombination are well-recognized mechanisms for the genetic variation and evolution of enteroviruses^25,26^. The lack of fidelity of the RNA-dependent RNA polymerase of these viruses results in high error rates through nucleotide mis-incorporation during genome replication, explaining how these viruses rapidly mutate and evolve^3,27^. However, CA16 is relatively genetically stable compared with EV71, with fewer CA16 than EV71 subgenotypes having been identified^21,26^. Viruses from different serotypes, genotypes, and subgenotypes of EV71 and CA16 have been reported to cocirculate and coinfect the same patients during epidemics of HFMD in the Asia-Pacific region^16,28^, with intratypic and intertypic genetic recombination among enteroviruses having been frequently reported^11,16,21,23,29–43^. Complete genome analyses revealed preferential recombination sites located in the nonstructural protein coding regions P2 (2A/2B junction) and P3 (3C and 3D regions), where nucleotide sequences with high similarities are present among EV-A^11,16,21,23,26,31–33^. The emergence of recombinant viruses, either recombined between strains within the EV71 serotype or between the EV71 and CA16 serotypes, have been postulated to potentially be responsible for large-scale HFMD outbreaks, and these strains are associated with higher virulence in mouse models and in in vitro assays^11,44,45^. The first evidence for intratypic and intertypic recombination in EV71 strains was reported during an outbreak in the late 1990s, which was subsequently followed by confirmatory reports from several countries in East and Southeast Asia^11,16,21,23,26,29–43^. In addition, the emergence of EV71 double- or triple-recombinant strains was reported during HFMD epidemics in 2008–2010^36,37,39^. In this study, the complete genome sequences of EV71 and CA16 isolates obtained between 2006 and 2014 in Thailand were analyzed for subgenotype and genetic recombination. Our results suggest that multiple introductions of recombinant viruses into Thailand are likely to have occurred.

## Results

### Enteroviral subgenotyping

The VP1 nucleotide sequences from 32 enteroviruses were phylogenetically analyzed together with reference sequences using MEGA version 5.0 (https://www.megasoftware.net). The viruses were identified as 25 EV71 isolates (comprising 20 B5, 1 C2, 2 C4a, and 2 C4b subgenotypes) and 7 CA16 isolates (comprising 6 B1a and 1 B1b subgenotypes). Notably, the dominant EV71 strains circulating between 2011 and 2014 belonged to subgenotype B5. The EV71 subgenotype C4b was first isolated in Thailand in 2006 in this study but was not detected in 2009, whereas the subgenotype C4a was first isolated in 2009 and currently persists in Thailand. CA16 isolates were less commonly detected than EV71 isolates. Of the seven CA16 isolates, six belonged to the B1a subgenotype and one belonged to the B1b subgenotype. Accession numbers for the VP1 nucleotide sequences of these viruses are shown in Table 1.

**Table 1:** List of the 32 enteroviruses, including 25 EV71 and 7 CA16 strains, used for phylogenetic tree construction and genetic recombination analyses

Virus	No.	Virus name	Year of isolation	GenBank accession no.		Subgenotype	No. of nucleotides analyzed
				VP1	Complete genome		
EV71	1	SI01/TH(NMA)/06	2006	EF203407	KX372308	C4b	7350
	2	Siriraj01/TH/08	2008	FJ862992	KX372309	C4b	7316
	3	SiICRC08/TH/2011	2011	JQ900607	KX372310	B5	7418
	4	SiICRC10/TH/2011	2011	JQ900605	KX372311	B5	7427
	5	SiICRC15/TH/2011	2011	JQ900613	KX372312	B5	7430
	6	SiICRC16/TH/2011	2011	JQ900604	KX372313	B5	7419
	7	SiICRC01/TH/2012	2012	KF748138	KX372314	B5	7416
	8	SiICRC02/TH/2012	2012	KF748139	KX372315	B5	7418
	9	SiICRC03/TH/2012	2012	KF748140	KX372316	B5	7418
	10	SiICRC04/TH/2012	2012	KF748133	KX372317	B5	7422
	11	SiICRC05/TH/2012	2012	KF748134	KX372318	B5	7417
	12	SiICRC06/TH/2012	2012	KF748135	KX372319	B5	7419
	13	SiICRC09/TH/2012	2012	KM675911	KX372320	B5	7414
	14	SiICRC10/TH/2012	2012	KM675912	KX372321	B5	7420
	15	SiICRC11/TH/2012	2012	KM675913	KX372322	B5	7417
	16	SiICRC01/TH/2013	2013	KF748136	KX372323	B5	7417
	17	SiICRC02/TH/2013	2013	KF748141	KX372324	C2	7409
	18	SilCRC03/TH/2013	2013	KM675907	KX372325	B5	7410
	19	SiICRC04/TH/2013	2013	KM675908	KX372326	B5	7378
	20	SiICRC05/TH/2013	2013	KM675915	KX372327	B5	7417
	21	SiICRC01/TH/2014	2014	KM675916	KX372328	C4a	7395
	22	SiICRC05/TH/2014	2014	KM675920	KX372329	B5	7418
	23	SiICRC06/TH/2014	2014	KM675921	KX372330	B5	7398
	24	SiICRC07/TH/2014	2014	KM675922	KX372331	B5	7421
	25	SiICRC08/TH/2014	2014	N/A	KX372332	C4a	7344
CA16	1	SiICRC04/TH/2011	2011	KF748145	KX372333	B1a	7411
	2	SiICRC05/TH/2011	2011	KF748146	KX372334	B1a	7422
	3	SiICRC06/TH/2011	2011	KF748147	KX372335	B1a	7418
	4	SiICRC01/TH/2012	2012	KF748137	KX372336	B1b	7416
	5	SiICRC02/TH/2012	2012	KM675923	KX372337	B1a	7395
	6	SiICRC03/TH/2012	2012	KM675924	KX372338	B1a	7371
	7	SiICRC01/TH/2014	2014	KM675925	KX372339	B1a	7415

*N/A* not applicable

The length of a genome encompasses from the first nucleotide in the 5ʹ-UTR to the last nucleotide in the 3ʹ-UTR and may include the poly(A) tail

### Genomic characterization of the enteroviral isolates

Among the 25 EV71 isolates, only 5 full-length genome sequences were obtained. Excluding the poly(A) tracts, the complete genomes of the four EV71-B5 isolates were 7412 nucleotides long, while that of an EV71-C2 subgenotype isolate was 7408 nucleotides long. The noncoding 5ʹ-UTRs were 747 nucleotides long, while the 3ʹ-UTRs preceding the poly(A) tail were 83 nucleotides long. The 5ʹ-UTRs and/or the 3ʹ-UTRs of the remaining 20 EV71 isolates were not completely sequenced, and genome sequence lengths of 7318–7409 nucleotides were determined. Based on analyses of the full-length genome sequences, the G + C content of the EV71 genomes varied from 47.95–48.31%, excluding the 3ʹ-poly(A) tail.

The complete genome sequence of a CA16 subgenotype B1a isolate was 7410 nucleotides long and was flanked by a 745-nucleotide 5ʹ-UTR and an 82-nucleotide 3ʹ-UTR preceding the poly(A) tail. The genome sequences of the remaining six isolates had lengths between 7371 and 7402 nucleotides. The G + C content of the complete CA16 genome was 47.4%, excluding the 3ʹ-poly(A) tail.

**Fig. 1:**
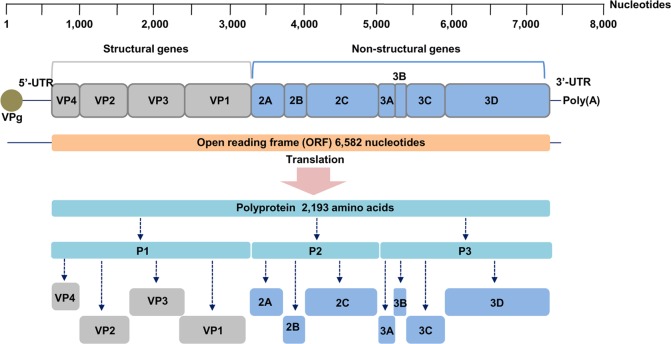
Enterovirus RNA genome organization and processing pattern of the polyprotein

All the EV71 and CA16 genomes contained a single open reading frame (ORF) of 6582 nucleotides encoding a polyprotein of 2193 amino acids as shown in Fig. 1. All genomic sequences have been deposited in the GenBank database with the accession numbers KX372308 to KX372339.

### Phylogenetic analyses of the enteroviral genomes

**Fig. 2:**
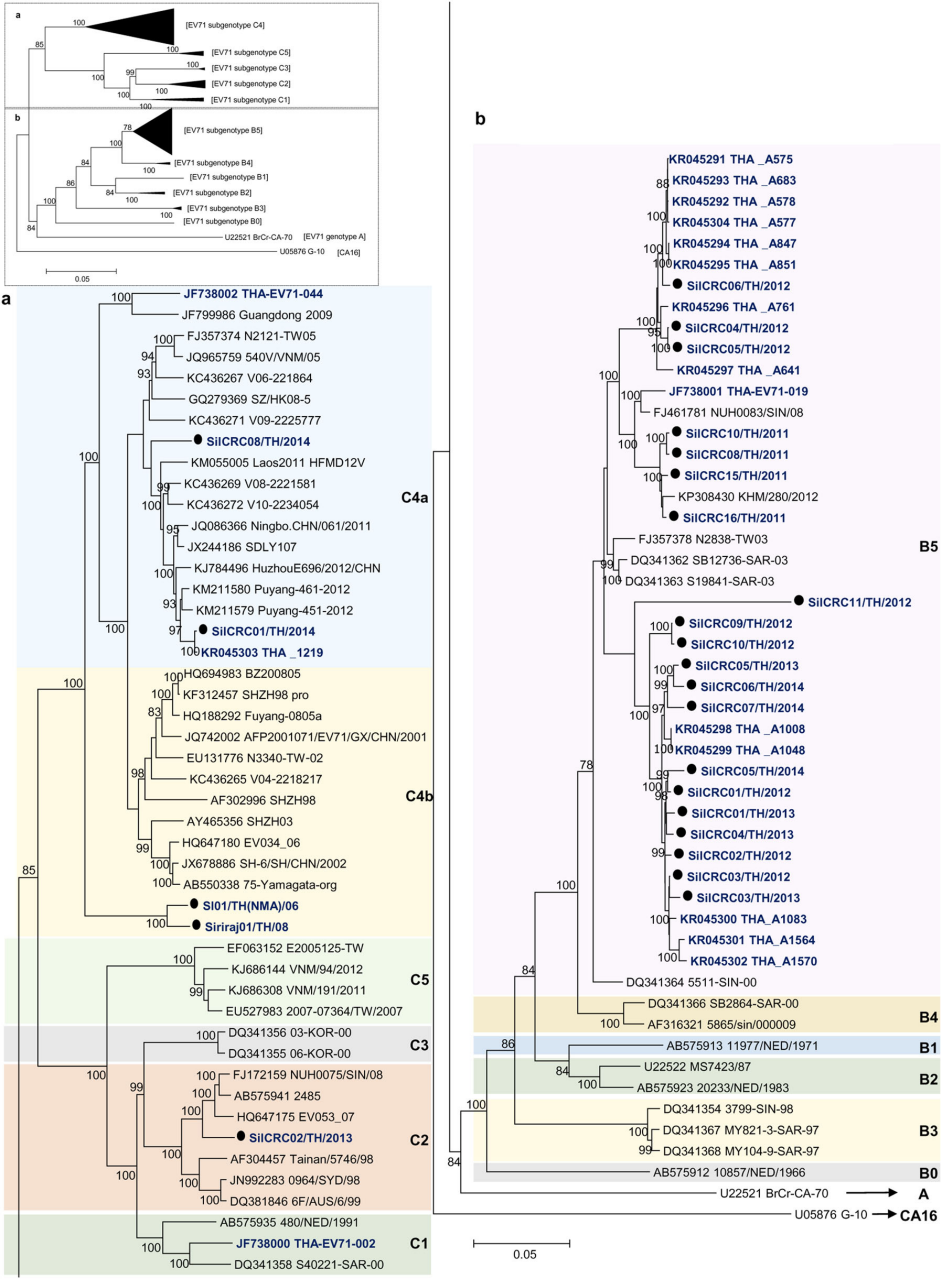
Subgenotyping of EV71 Thai isolates. The phylogenetic tree was extended for **a** EV71 genotype C and **b** EV71 genotype B, based on complete genome sequences. The phylogenetic dendrogram was constructed by the neighbor-joining method on the basis of the maximum composition likelihood model using MEGA 5.0. The prototype CA16 G-10 strain was used as an outgroup. Bootstrap values of 1000 replicates are shown at the branch points. EV71 Thai isolates are indicated in blue letters and strains marked by black circles were from this study

**Fig. 3:**
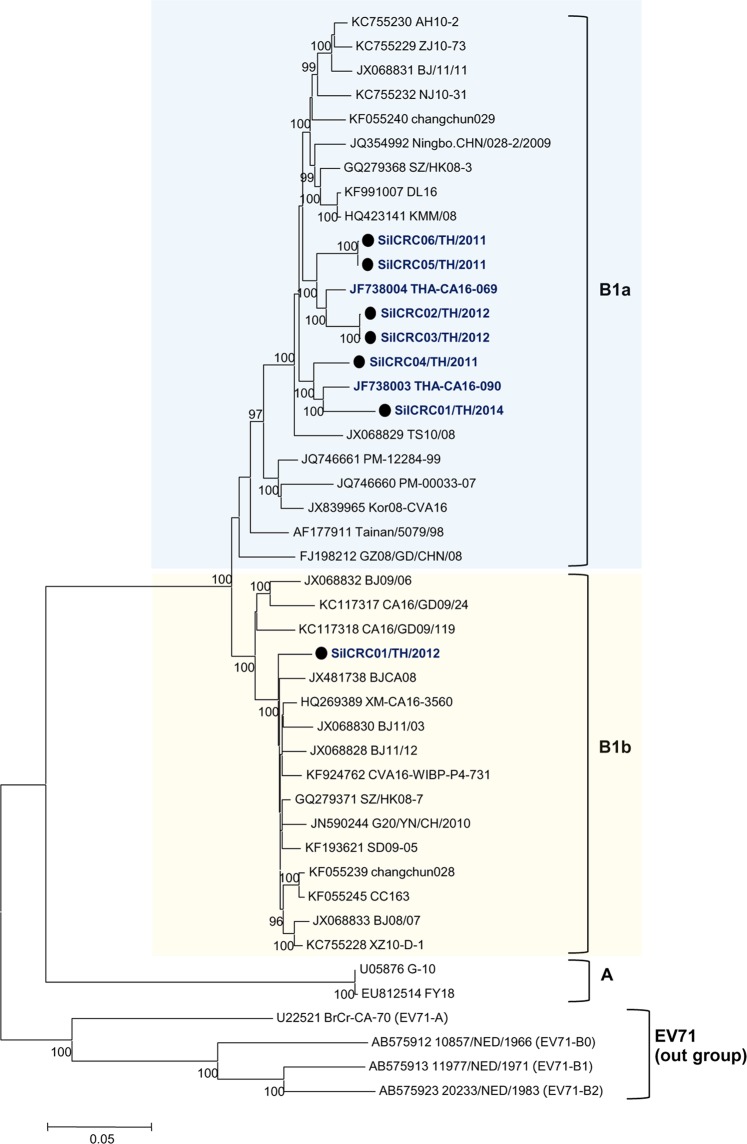
Subgenotyping of CA16 Thai isolates based on complete genome sequences. The phylogenetic dendrogram was constructed by the neighbor-joining method on the basis of the maximum composition likelihood model using MEGA 5.0. The prototype EV71 genotypes A and B were used outgroups. Bootstrap values of 1000 replicates are shown at the branch points. CA16 Thai isolates are indicated in blue letters and strains marked by black circles were from this study

Phylogenetic trees were constructed based on the nucleotide sequences comprising the 5ʹ-UTR and the P1 (VP1–VP4), P2 (2A–2C), and P3 (3A–3D) regions. The nucleotide sequences of the Thai EV71 and CA16 isolates were analyzed for their genetic relationships with the reference subgenotypes, EV-A prototypes and the oldest available strains^41^ available in the GenBank database using MEGA 5.0. All of the enterovirus isolates assayed in this study clustered together with their prototypes and the oldest available strains EV-A strains, as shown in Fig. 2 and Supplementary Figure S1. The results identified the 25 EV71 isolates as 20 B5, 2 C4a, 2 C4b, and 1 C2 subgenotypes, while among the seven CA16 isolates, six isolates belonged to the B1a subgenotype while one was a B1b subgenotype as shown in Fig. 3. A subgenotyping analysis based on the VP1 region or the entire genome yielded the same result.

### Analyses for genetic recombination in the viral genomes

The viral genome regions of the study viruses, including the 5ʹ-UTR and the P1, P2, and P3 regions, were phylogenetically analyzed for genetic relationships with the other enteroviral genomes. Similarity plots and bootscan analyses were performed to identify potential genetic recombination sites in the viral genomes using various prototypes and the oldest available EV-A strains as the reference sequences (Supplementary Table S3).

**Fig. 4:**
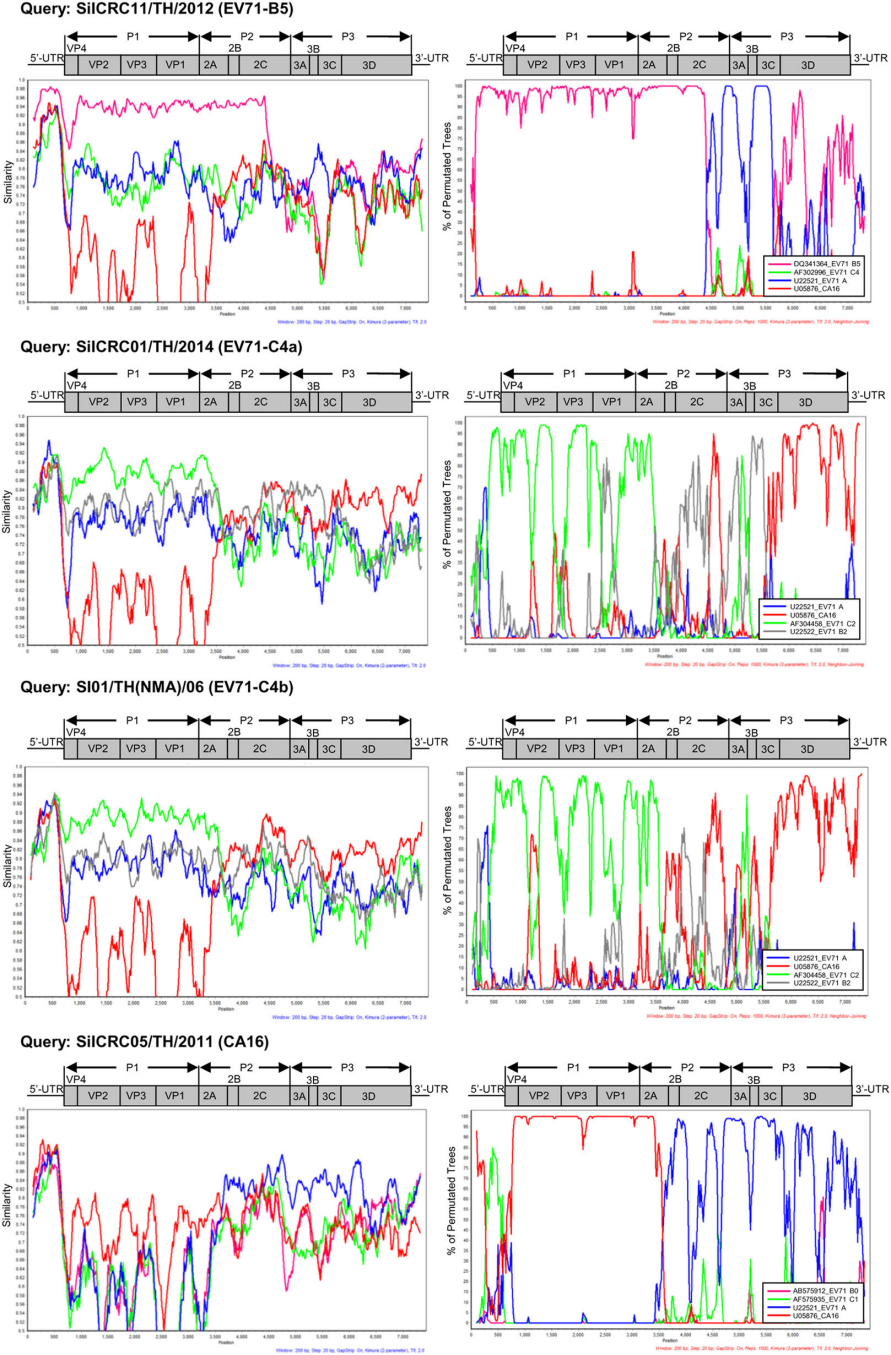
Similarity plots and bootscan analyses of EV71 (B5 and C4 subgenotypes) and CA16 on the basis of complete genomes using SimPlot. The similarity was calculated in a sliding window size of 200 nucleotides using the Kimura 2-parameter distance method. Bootscan analysis was performed using the neighbor-joining tree model and the Kimura 2-parameter distance algorithm with a window size of 200 nucleotides

Phylogenetic tree analysis of each viral genome region showed that 19 of 20 EV71-B5 Thai isolates clustered together using the 5511-SIN-00 EV71-B5 prototype as the reference strain (accession number DQ341364). The highest degree of similarity to the reference nucleotide sequence suggested that these 19 EV71-B5 Thai isolates were nonrecombinant strains. In contrast, the P3 region of an EV71-B5 strain, SiICRC11/TH/2012, was more evolutionary distant and was segregated from the reference strain. Similarity plot and bootscan analyses showed the recombination breakpoint at the P2–P3 region at approximate nucleotide positions of 4500 and 5800, suggesting intratypic recombination with EV71 genotype A or intertypic recombination with the other EV-A members as shown in Fig. 4 and Supplementary Figures S2 and S3. In addition, phylogenetic analysis showed that the EV71-C2 isolate SiICRC02/TH/2013 was not a recombinant virus and was closely related to the oldest identified strain Tainan/5746/98 (GenBank accession no. AF304457). In contrast, the two EV71-C4a isolates (SiICRC01/TH/2014 and SiICRC08/TH/2014) and the two EV71-C4b (SI01/TH(NMA)/06 and Siriraj01/TH/08) isolates were closely related to SHZH98, the oldest identified C4 isolate and a previously known recombinant virus^29^, as shown in Supplementary Figures S1 and S4. A phylogenetic tree of the P1 regions showed that all four EV71-C4 Thai isolates resembled the EV71 genotype C, while the P2 regions resembled EV71 genotype B and the P3 regions resembled the other EV-A members (CA4, CA14, and CA16) (Supplementary Figures S2 and S3). The phylogenetic analysis results agreed well with both the similarity plot and bootscan analyses, which demonstrated the occurrence of double recombination with two breakpoints in the P2 (resembling the EV71 genotype B: MS/7423/87) and the P3 (resembling CA4, CA14, and CA16) regions. This type of double recombination was observed in all EV71-C4 isolates as shown in Fig. 4 and Supplementary Figures S2–S5. The results are summarized in Table 2.

**Table 2:** Summary of genetic recombination events among the enterovirus isolates circulating in Thailand between 2006 and 2014

Virus	Subgenotype	No. of analyzed isolate	No. of recombinant detected (%)	Name of recombinant strain	Recombination detection by SimPlot program
EV71	B5	20	1 (5%)	SiICRC11/TH/2012	Some part of the P2-P3 region resembled EV71 genotype A, and serotypes CA3, CA6, and CA12
	C4a	2	2 (100%)	SiICRC01/TH/2014 SiICRC08/TH/2014	P2 resembled EV71 genotype B P3 resembled serotypes CA4, CA14, and CA16
	C4b	2	2 (100%)	SI01/TH(NMA)/06 Siriraj01/TH/08	P2 resembled EV71 genotype B P3 resembled serotypes CA4, CA14, and CA16
CA16	B1a	6	6 (100%)	SiICRC04/TH/2011 SiICRC05/TH/2011 SiICRC06/TH/2011 SiICRC02/TH/2012 SiICRC03/TH/2012 SiICRC01/TH/2014	P2-P3 region resembled EV71 genotype A, and serotypes CA3, CA6, and CA12
	B1b	1	1 (100%)	SiICRC01/TH/2012	P2-P3 region resembled EV71 genotype A, and serotypes CA3, CA6, and CA12

Similarly, the phylogenetic tree, similarity plot and bootscan analyses showed that all seven CA16 strains studied were also recombinant viruses. The P1 region of the viral genomes showed the highest similarity to the CA16 prototype, but the P2 and P3 regions resembled several enteroviruses, including EV71 genotypes A, CA3, CA6, and CA12, suggesting intertypic recombination events. The recombination breakpoints were identified at the 2A–2B junctions (Fig. 4, Supplementary Figures S2–S6 and Table 2).

### Analysis of viral genome identity

The genomic sequences of the study viruses were comprehensively analyzed by region, i.e., the 5ʹ-UTR and the P1, VP4, VP2, VP3, VP1, P2, 2A, 2B, 2C, P3, 3A, 3B, 3C, and 3D regions, for identity to the oldest representative strains or prototypes, including EV71-A, EV71-B5, EV71-C2, CA3, CA4, CA6, CA12, CA14, and CA16, as shown in Supplementary Table S4. The nucleotide sequences of 19 nonrecombinant EV71-B5 isolates perfectly matched the reference EV71-B5 strain 5511-SIN-00 for all the analyzed regions, while the recombinant EV71-B5 isolate (SiICRC11/TH/2012) shared high nucleotide sequence identity to the reference EV71-B5, except for the sequences at the recombination breakpoints (the P3, 3A, 3B, and 3C regions) which were closer to EV71-A, CA3, CA6, and CA12. The nucleotide sequences of the EV71-C4a and C4b isolates showed high sequence identities to the reference EV71 genotype C strain Tainan/5746/98, except for the sequences at the recombination breakpoints (the P2, 2B, 2C, 3A, 3B, 3C, and 3D regions) which were closer to EV71-B5, CA4, CA14, and CA16. Similarly, all of the seven CA16 isolates assayed in this study shared a high degree of homology to the G-10 prototype strain, except for the sequences at the recombination breakpoints (the P2, 2B, 2C, P3, 3A, 3B, 3C, and 3D regions) which showed higher similarity to EV71 A, CA3, CA6, and CA12.

## Discussion

Multiple subgenotypes of EV71 have circulated in the Thai population during the past two decades, starting with B4 and C1 in 2001 and followed by C5 in 2006, C4b in 2006, B5 and C2 in 2007, and C4a in 2009 (information obtained from the GenBank database sequences deposited by our laboratory and others)^21,46–49^. Subgenotype replacement was observed over time, but the exact times of their emergence and extinction are not known in most cases. Nevertheless, this study identified SI01/TH(NMA)/06 as the first C4b strain introduced into Thailand in 2006, a virus that was isolated from a child who died of encephalitis. Molecular epidemiology and field investigations suggested that this strain originated from Shenzhen, China^46^. Our surveillance suggested that the subgenotype C4b disappeared after 2008. An analysis of the EV71 strain THA-EV71-044 (GenBank accession no. JF738002) documented in 2009 revealed it to be the first C4a subgenotype ever reported in Thailand^48^. While the circulating EV71 strains belong to multiple EV71 subgenotypes, the CA16 strains circulating in Thailand were primarily confined to subgenotype B1a. Only one CA16 B1b isolate, SiICRC01/TH/2012, was detected in 2012. Several other HFMD-associated enteroviruses have occasionally been isolated by our laboratory, such as coxsackieviruses A4 (CA4), CA6, CB1, CB4, and CB5 and echovirus 19. From 2012 to 2015, CA6 caused a large outbreak of mild HFMD in several countries, including Thailand, and became the dominant subgenotype for a few years^49,50^. At present, the most common HFMD-associated viruses are EV71 followed by CA16 and CA6 (unpublished data).

In the present study, a complete genome analysis of 25 EV71 and 7 CA16 strains isolated between 2006 and 2014 was performed to assess possible genetic recombination within the viral genomes. In general, the VP1 sequence has been used to subgenotype enteroviruses^51^. Subgenotypic identification based on VP1 or entire genome sequences, as was performed in this study and by others, yielded the same results^52^. The full-length genome and the genomic organization of both EV71 and CA16 viruses in this study were similar to those of EV71 and CA16 strains that were previously published or deposited in the GenBank database^23,42,53^, although slight differences in the lengths of the 5ʹ-UTRs and 3ʹ-UTRs were observed. Our study did not observe any insertions or deletions in the protein encoding regions (P1, P2, and P3), suggesting that the viruses contained conserved functional proteins with no frameshift mutations. However, nucleotide substitutions were scattered across the genomes, suggesting that genetic variations among EV71 and CA16 strains are common. Based on the complete genomic sequences, our phylogenetic trees demonstrated that all 32 enteroviral isolates were perfectly clustered with the prototypes and reference strains. Our viral isolates exhibited an evolutionary relationship with those previously reported from China, Japan, and many countries in Southeast Asia^15,32,43,54^. The cocirculation of EV71 and CA16 in the same HFMD outbreak is common and has been observed in various countries^13,16,28^, including Thailand^47,48^.

The oldest identified EV71-B5 strain (5511-SIN-00) was identified in 2000 as a nonrecombinant virus in Singapore. Evidence of genetic recombination in EV71-B5 was first reported in Chongqing City, China, in 2014 in the viral strain CQ2014-86/CQ/CHN/2014 (accession number KU647000), and the recombination breakpoints were in the 5ʹ-UTR and in the P2-P3 region^32^. Interestingly, genetic recombination in the EV71-B5 isolate assayed in this study was discovered in 2012. Similarity plot and bootscan analyses revealed that SiICRC11/TH/2012 was the only isolate among the 20 assayed EV71-B5 isolates with the potential for intratypic or intertypic recombination in the 2C region between the nucleotide positions 4500 and 5800, resembling the EV71 genotypes A, CA3, CA6, and CA12. Moreover, the results of our study suggested that all of our two EV71-C4a and two EV71-C4b isolates were double recombinants, with two recombination breakpoints in the 2A–2B junction and the 3C–3D region. Our analysis also showed that this recombination pattern was also present in THA_A1219, an EV71-C4 strain isolated in 2014 by another group of Thai investigators^43^.

We showed that six out of seven CA16 strains in this study belong to subgenotype B1a, with only one strain belonging to subgenotype B1b. The CA16 recombinant viruses may have circulated in Thailand for specific period of time as has occurred in China^16,23,30^. The results of similarity plot and bootscan analyses demonstrated multiple intertypic recombinations in all of these seven CA16 strains such that the P1 region resembled the CA16-G10 prototype, but the P2–P3 regions resembled the EV71 genotypes A, CA3, CA6, and CA12. Intertypic recombination in CA16 viruses has been previously reported in mainland China^16,23,30^. The recombination breakpoint was frequently detected in the P2–P3 regions, but not in the P1 region, which encodes the VP1–VP4 structural proteins. This finding suggests that the nonstructural proteins of enterovirus members are highly conserved and are interchangeable.

At present, HFMD is not only confined to the Asia-Pacific region. HFMD epidemics associated with subgenotypes other than B5 and C4 (primarily from C1 and C2) have been reported from several countries in Europe and North America^3,4,21,55–58^. Genetic recombination was also observed in those viruses. However, HFMD associated with recombinant EV71 or C16 viruses is more common in Asian countries, although the role of these recombinant viruses in public health is uncertain. Clinical manifestations in patients infected with recombinant and nonrecombinant EV71 isolates were comparable, suggesting that the virulence of these types of strains might not be different^41^. In contrast, the results of viral phenotype studies in cell culture- and mouse model-based systems suggested that the recombinant viruses are likely more virulent^44,45^. The role of recombinant viruses with respect to the magnitude of an HFMD outbreak is also controversial. A large outbreak involving 6049 HFMD cases in Fuyang, China, was caused by EV71-C4a together with a small number of intertypic recombinant EV71-C4a viruses exhibiting CA16 G-10 sequences in the 3D region^11^. Similarly, a small number of intratypic EV71-C4a recombinant viruses with EV71-C4b sequences at the 2B–2C region were isolated in mainland China during a very large outbreak between 2011 and 2012 that involved millions of HFMD cases^16^. The HFMD outbreak in Cambodia in 2012, which resulted in 61 cases with 56 deaths, was primarily caused by EV71-C4a and few EV71-B5 subgenotypes^15^.

The impact of the emergence of recombinant viruses in HFMD will require a great deal of observation. The recombinant EV71-C4a strain was predominantly found in China in 2008^11^, and is currently the major circulating virus. Subsequently, this recombinant virus may have spread to several other countries. EV71-B5 has become the predominant subgenotype associated with HFMD in Thailand in recent years. The role of the EV71-B5 recombinant virus on the spread of HFMD and clinical outcomes should be further explored. Moreover, surveillance for circulating strains and subgenotype replacement are also important in terms of vaccine selection and development.

## Materials and methods

### Ethics issue

This study was approved by the Siriraj Hospital Institutional Review Board, Faculty of Medicine, Siriraj Hospital, Mahidol University, with the certificate of approval number Si058/2013 for the protocol number 689/2555(EC3).

### Cell line

Vero cells (ATCC, catalog no. CCL-81) derived from African green monkey kidney were cultured in Earle’s minimal essential medium (EMEM) supplemented with 10% fetal bovine serum (FBS; Gibco, NY) penicillin, gentamycin, and fungizone. The cell monolayers used for viral isolation and propagation were maintained in media supplemented with 2% FBS.

### Study viruses

Throat swabs and fecal specimens were collected between 2006 and 2014 from various provinces of Thailand, and a total of 32 enteroviruses were isolated from 31 pediatric patients (30 HFMD and 1 encephalitis case) and 1 asymptomatic adult with a history of contact with an HFMD patient. Information for some patients has been reported previously^46,47^. The viruses were grown for 2 or 3 passages before being subjected to nucleotide sequencing.

### Polymerase chain reaction (PCR) and complete genome sequencing

Culture supernatants of enterovirus-infected Vero cells were extracted for total RNA using a QIAamp^®^ Viral RNA mini kit (Qiagen, GmbH, Hilden, Germany). The viral RNA was amplified in eight DNA fragments that spanned the entire genome (~7.4 kilobases (kb) long). Each DNA fragment was sequenced directly using a panel of sequencing primers designed by our group and previous investigators^53^ as listed in Supplementary Tables S1 and S2.

One step RT-PCR kits (Qiagen GmbH, Hilden, Germany) were used for viral RNA amplification following the manufacturer’s instructions. A 50 μl reaction mixture was subjected to reverse transcription at 50 °C for 30 min, followed by heat inactivation of the reverse transcriptase enzyme together with activation of DNA polymerase at 95 °C for 15 min that was followed by 35 cycles of denaturation at 94 °C for 45 s, primer annealing at 48–55°C for 45 s and extension at 72 °C for 2 min. The reaction was completed with a final extension step at 72 °C for 10 min. The PCR DNA products were electrophoresed, purified using a QIAquick^®^ gel extraction kit (Qiagen), and were sequenced by Macrogen Inc., Seoul, Korea (http://dna.macrogen.com/eng/). Bidirectional sequences were obtained and assembled using BioEdit Sequence Alignment Editor version 7.0.4.1^59^. Overlapping DNA sequences with at least 85% homology and a minimum of 20 nucleotides of overlap were CAP contiged to generate consensus sequences.

### Nucleotide sequencing and phylogenetic tree construction

Nucleotide sequences of the viral genomes and the deduced amino acid sequences were analyzed using BioEdit and the Basic Local Alignment Search Tool (BLAST) available at the U.S. National Center of Biotechnology Information (NCBI). The complete nucleotide sequences of all 32 enterovirus strains have been deposited in the GenBank database (www.ncbi.nlm.nih.gov) under accession numbers KX372308 to KX372332 for EV71 and KX372333 to KX372339 for CA16, as shown in Table 1.

The nucleotide sequences encompassing the entire genomes or particular regions (5ʹ-UTR, P1, VP4, VP2, VP3, VP1, P2, 2A, 2B, 2C, P3, 3A, 3B, 3C, or 3D) were aligned with reference sequences retrieved from the GenBank database using the ClustalW multiple alignment application. Phylogenetic trees were constructed using MEGA version 5.0 (https://www.megasoftware.net/)^60^. The evolutionary distances were estimated using the neighbor-joining method and a maximum composite likelihood algorithm. The reliability of the neighbor-joining tree was estimated by a bootstrapping analysis using 1000 replicate datasets. The reference sequences of CA16 were used as outgroups for EV71 phylogenetic tree construction, while the EV71 genotypes A and B were used as the outgroups for CA16 phylogenetic tree construction. Bootstrap values of greater than 80% supported the tree topology, as shown at the nodes of each cluster.

### Genetic recombination analysis

The 32 enteroviral genomes assayed in this study were investigated for intratypic and intertypic genetic recombination. The genomic sequences were aligned with the prototypes and the oldest available strains of the species *Enterovirus A* (EV-A) using the ClustalW multiple alignment application in BioEdit. Sequences of the reference viral genomes were retrieved from the GenBank database using the accession numbers listed in Supplementary Table S3.

Similarity plot and bootscan analyses were performed using SimPlot (Similarity Plotting) version 3.5.1 (Stuart Ray, Johns Hopkins University, Baltimore, MD) (http://sray.med.som.jhmi.edu/SCRoftware/simplot/)^61^ using the default parameter settings. All gaps were removed from the DNA sequences, and ≥ 50% nongaps were required to analyze a set of data points. Bootscan analysis was performed using the neighbor-joining tree model and the Kimura 2-parameter distance algorithm with a window size of 200 nucleotides moving along the alignment in increments of 20 nucleotides with 1000 resampling. The use of the PHYLIP internal code (v3.5) and a 70% parental threshold were selected for notification if a region of recombination was detected. The nucleotide positions were indicated along the *x*-axis, and the sequence similarities or percentages of permutated trees were indicated along the *y*-axis.

## Supplementary information

Supplementary Figure S6

Supplementary Table S1

Supplementary Table S2

Supplementary Table S3

Supplementary Table S4

Supplementary Figure S1

Supplementary Figure S2

Supplementary Figure S3

Supplementary Figure S4

Supplementary Figure S5
