# Supplementary figures and images for: Complete genome analysis demonstrates multiple introductions of enterovirus 71 and coxsackievirus A16 recombinant strains into Thailand during the past decade
Source: Emerg Microbes Infect. 2018 Dec 14;7:214. doi: 10.1038/s41426-018-0215-x (PMC6294798; doi:10.1038/s41426-018-0215-x)

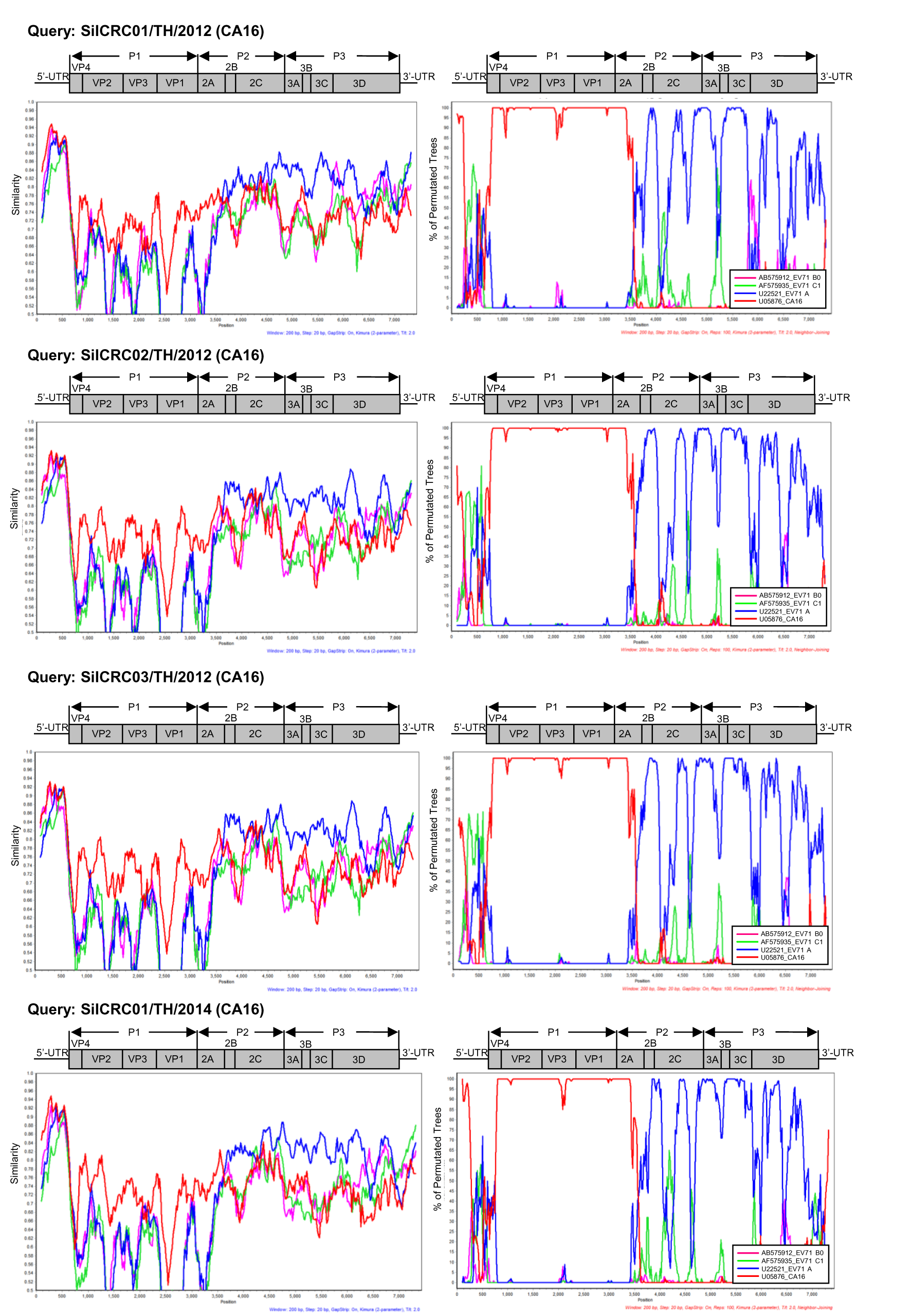

Supplement: Supplementary file 1 — Supplementary Figure S6 [file 41426_2018_215_MOESM1_ESM.tif]

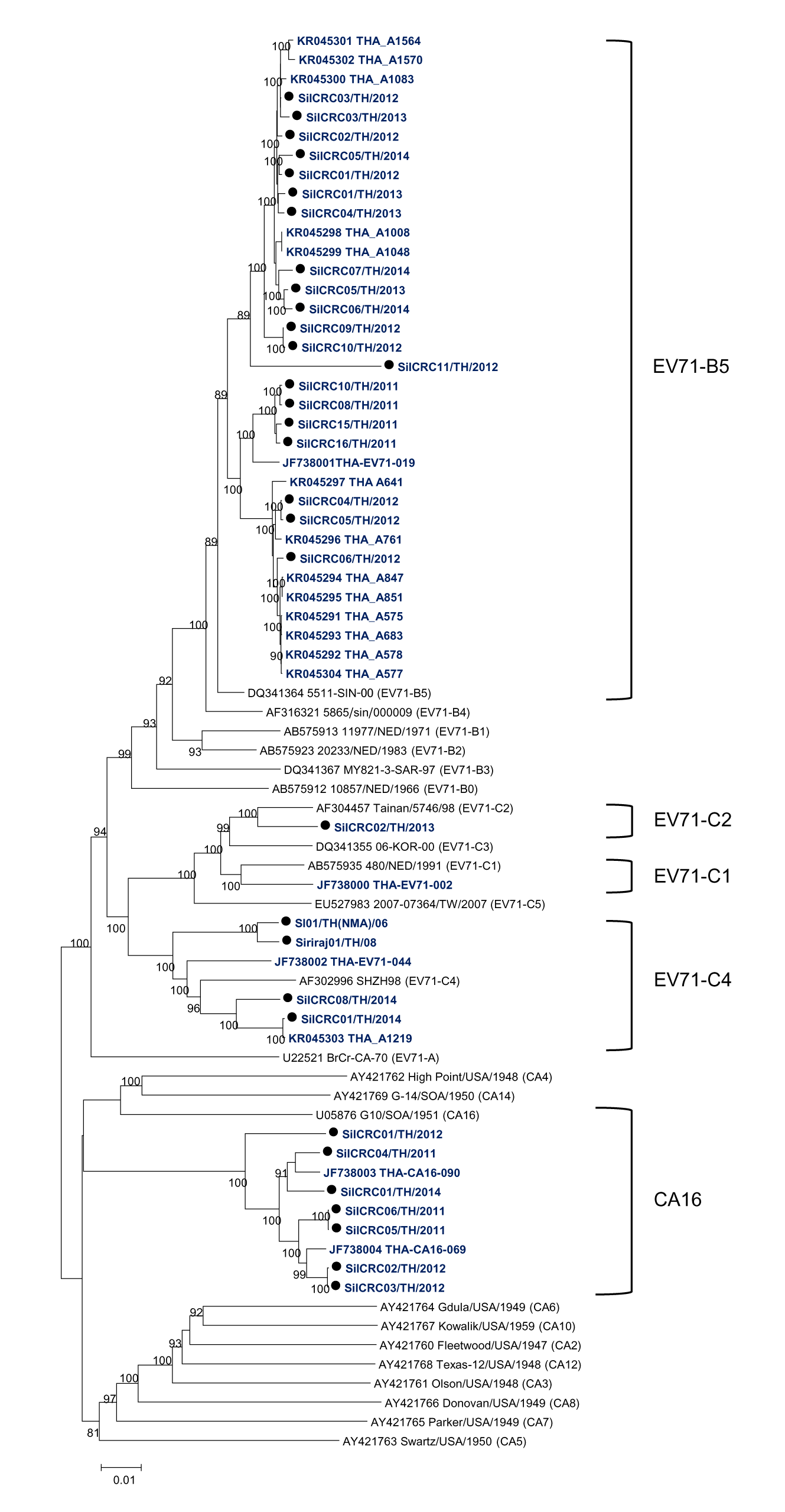

Supplement: Supplementary file 6 — Supplementary Figure S1 [file 41426_2018_215_MOESM6_ESM.tif]

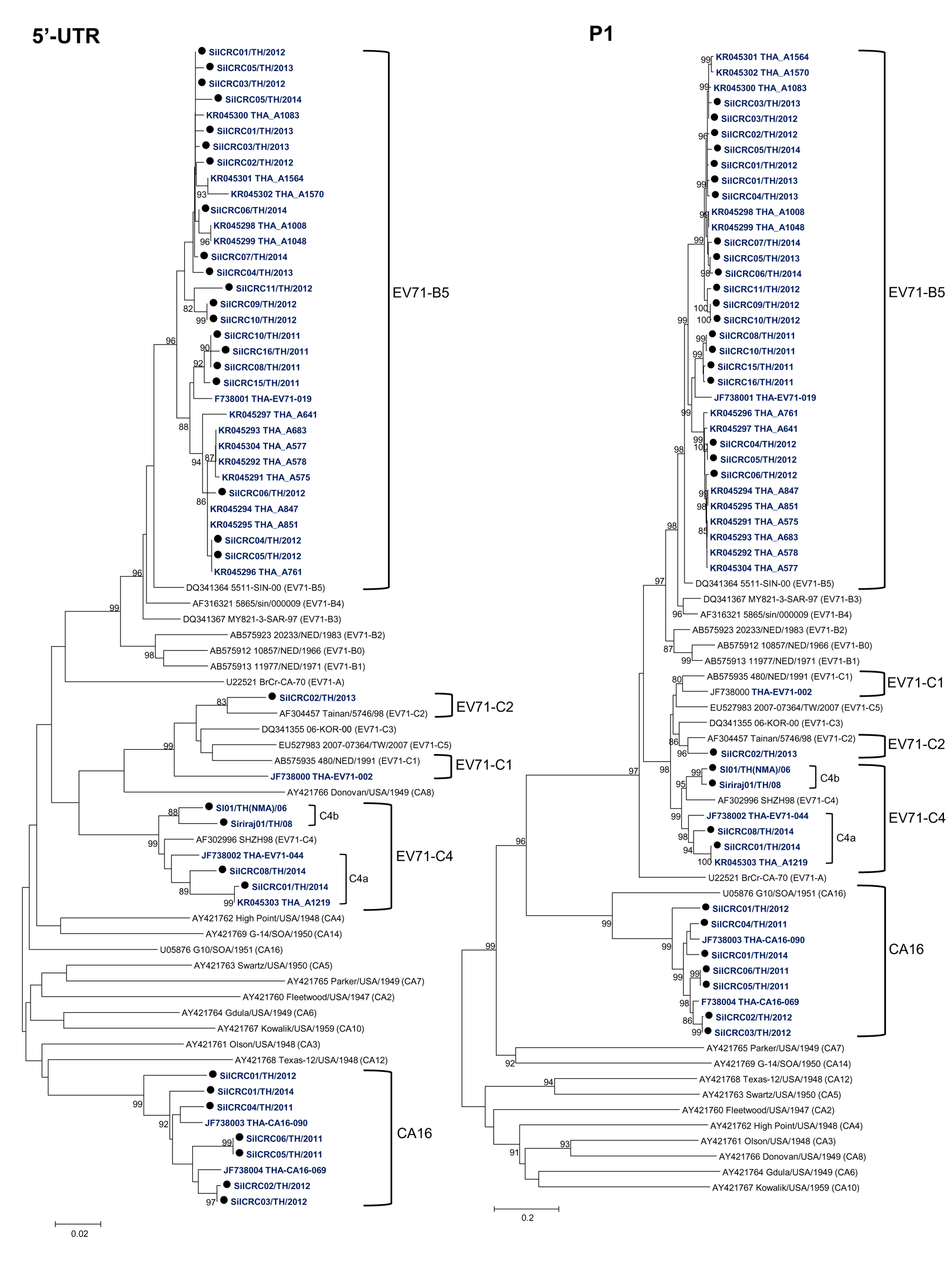

Supplement: Supplementary file 7 — Supplementary Figure S2 [file 41426_2018_215_MOESM7_ESM.tif]

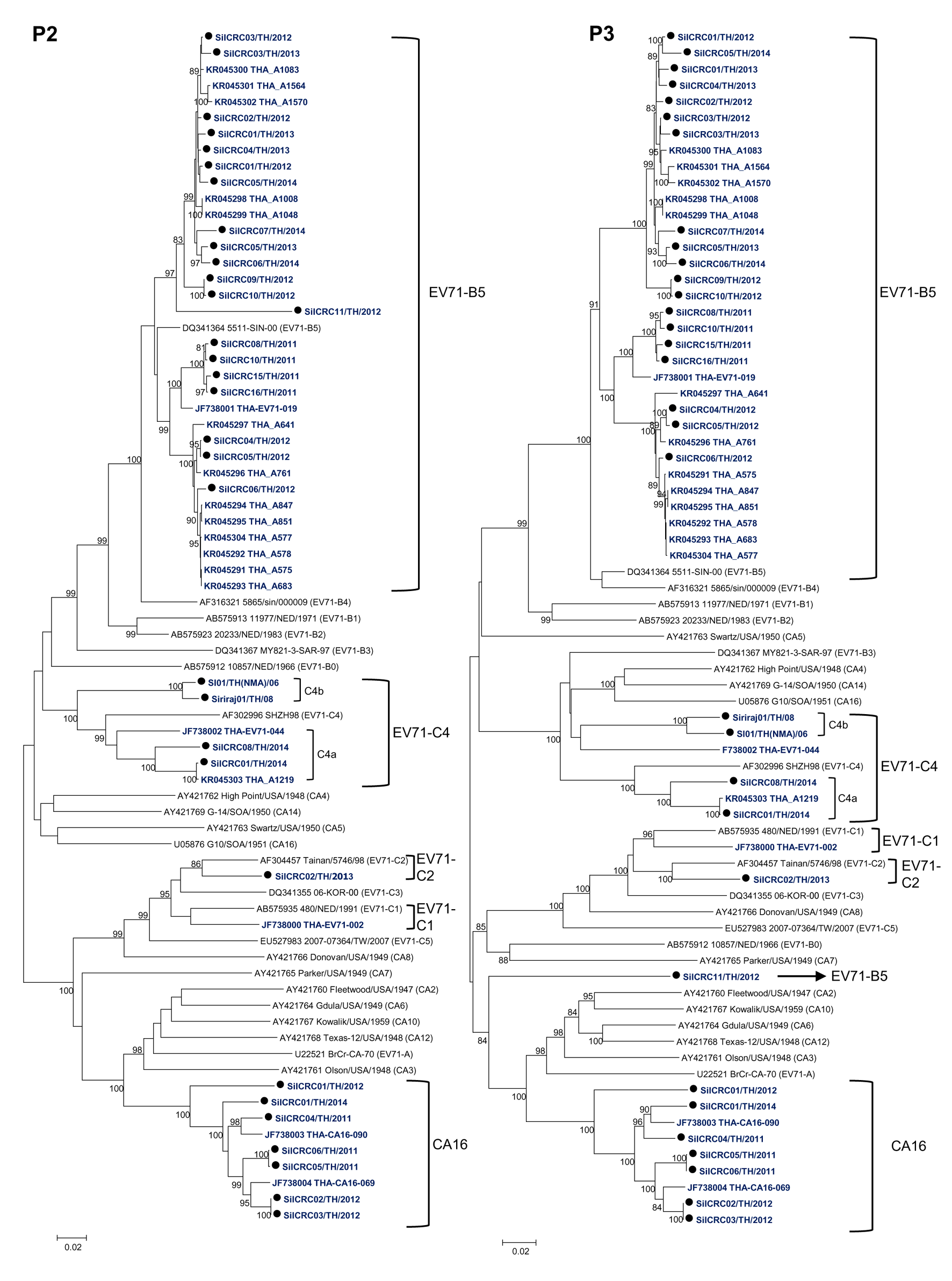

Supplement: Supplementary file 8 — Supplementary Figure S3 [file 41426_2018_215_MOESM8_ESM.tif]

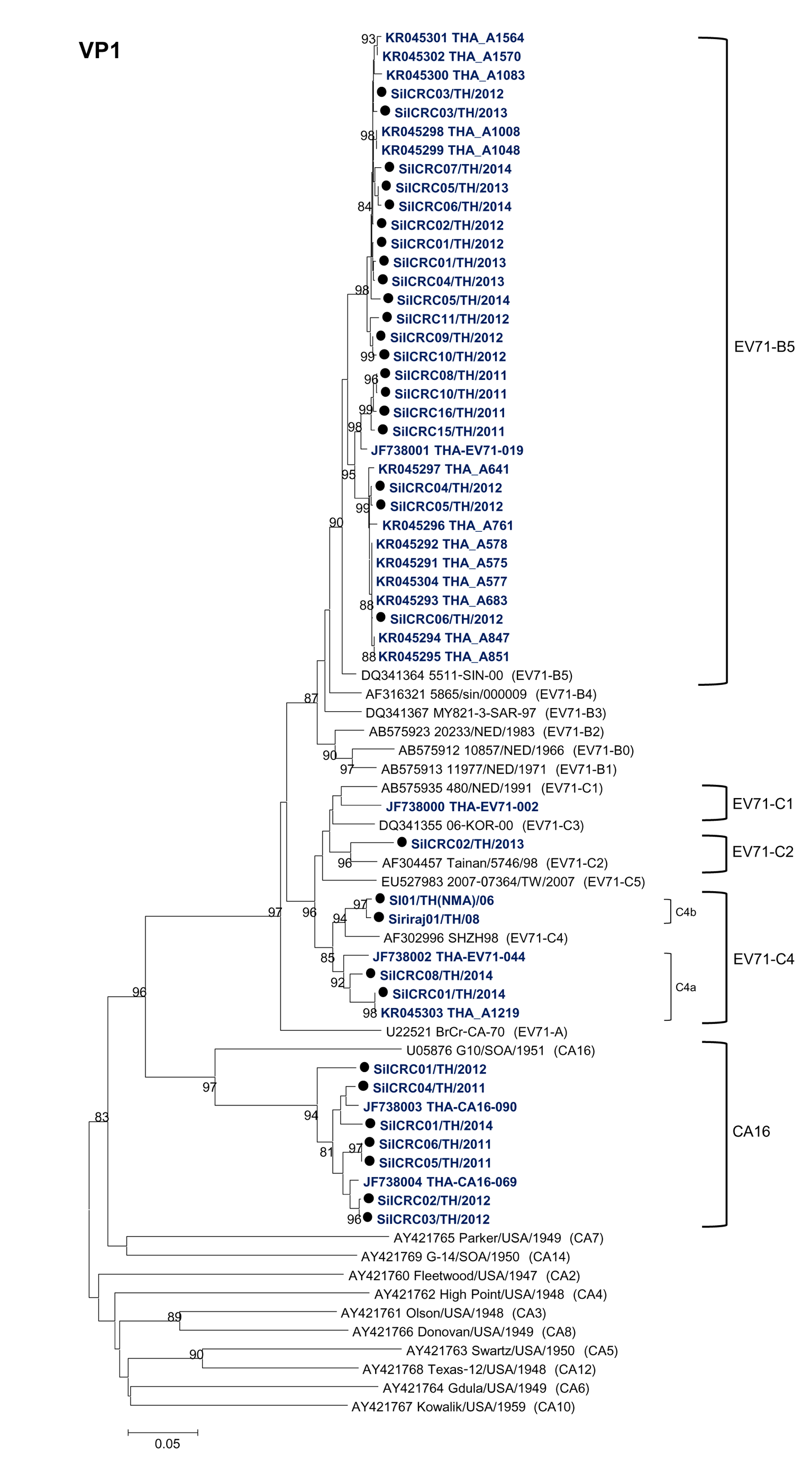

Supplement: Supplementary file 9 — Supplementary Figure S4 [file 41426_2018_215_MOESM9_ESM.tif]

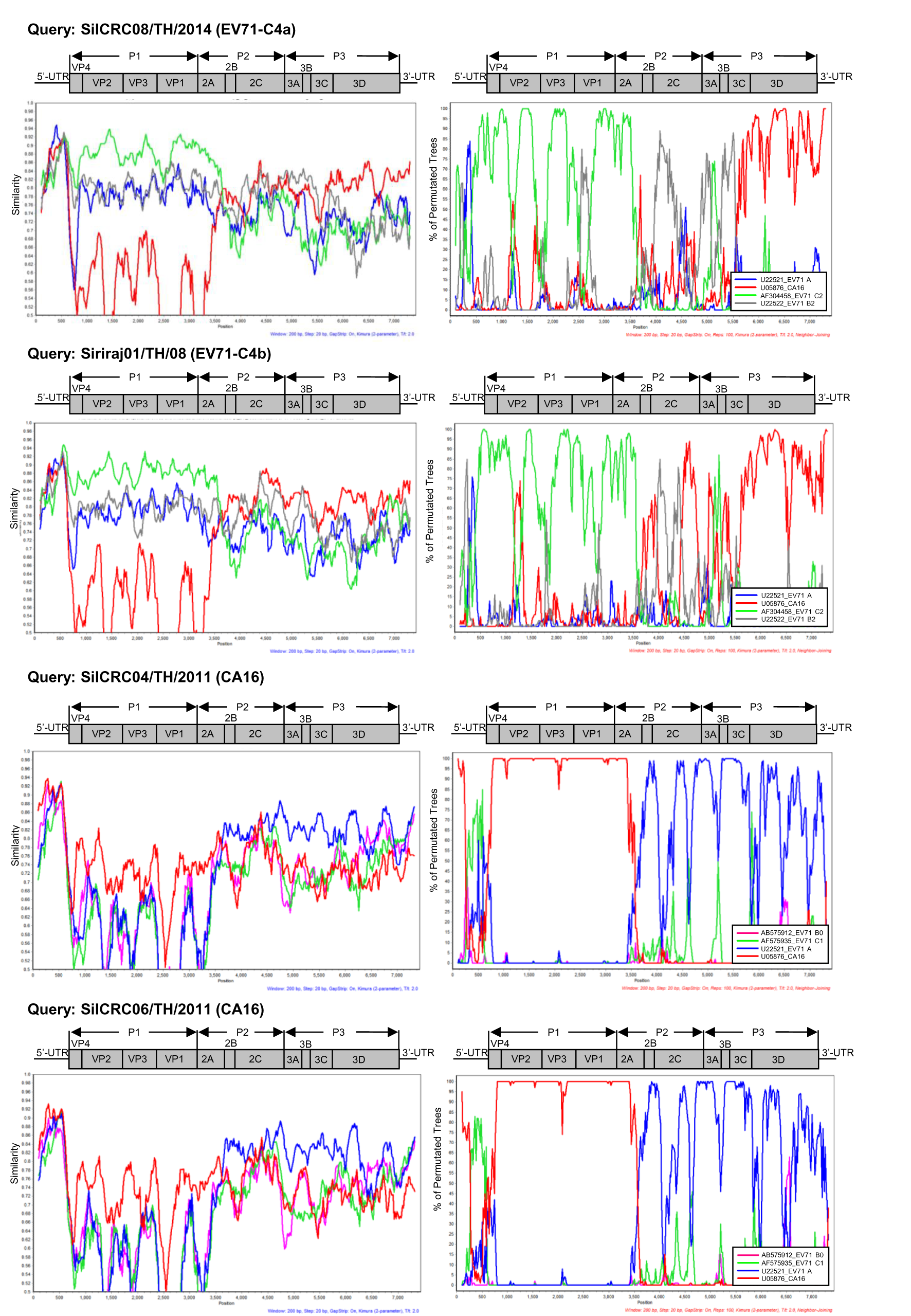

Supplement: Supplementary file 10 — Supplementary Figure S5 [file 41426_2018_215_MOESM10_ESM.tif]
