# Supplementary material for: Complete genome analysis demonstrates multiple introductions of enterovirus 71 and coxsackievirus A16 recombinant strains into Thailand during the past decade
Source: Emerg Microbes Infect. 2018 Dec 14;7:214. doi: 10.1038/s41426-018-0215-x (PMC6294798; doi:10.1038/s41426-018-0215-x)
Supplement: Supplementary file 2 — Supplementary Table S1 [file 41426_2018_215_MOESM2_ESM.doc]

**Supplementary Table S1.** Panel of amplification and sequencing primers for EV71

| **Fragment no.** | **Primer name** | **Nucleotide position** | **Sequence of primers**  **(5’ --->3’)** | **Product size (bp)** | **Annealing temperature** |
| --- | --- | --- | --- | --- | --- |
| 1 | FL-F-1C | 1-20 | TTAAAACAGCCTGTGGGTTG | ~1,075 | 52°C |
|  | 1075R | 1061-1080 | CAGTAMGAYGGCCARTCKCC |  |  |
|  | 636R | 636-616 | CCAATCCAATAGCTATATGGC |  |  |
| 2 | 447F | 447-467 | TAGTCCTCCGGCCCCTGAAT | ~920 | 55°C |
|  | 1366R | 1349-1367 | CGCCTGCCACTGTCCCWAT |  |  |
| 3 | 1178F | 1181-1198 | TCCAAGGGRTGGTAYTGG | ~1,200 | 48°C |
|  | 2376R | 2357-2376 | GCYGCCGCYAGTGCTATTAT |  |  |
| 4 | 2191F | 2192-2210 | TTTGGGCTRCAATCRTCTG | ~1,255 | 50°C |
|  | 3446R | 3428-3446 | GRGAGCTGTCTTCCCAAAC |  |  |
| 5 | 3200F | 3200-3219 | ATGAGAATGAAGCAYGTCAG | ~1,240 | 50°C |
|  | 4434R | 4418-4437 | CGGTGTTTGCTCTTGAACTG |  |  |
| 6 | 4016F | 4012-4029 | TGGNAGTCCYTGGGCTTG | ~1,170 | 55°C |
|  | 5182R | 5168-5184 | CCCTRCAGTAYTGGCGCACYTC |  |  |
| 7 | 4930F | 4926-4946 | GCCCHYTAGTGTGTGGGAARG | ~1,300 | 50°C |
|  | 6322R | 6224-6243 | CAVGCYTCYTCCATGCTCAT |  |  |
| 8 | 6239F | 6223-6239 | ATGAGCATGGARGADGC | ~1,180 | 50°C |
|  | FL-R | 7396-7428 | TTTTTTTTTTTTGCTATTYTGGTTATAACAAAT |  |  |
|  | 6792F | 6794-6810 | CTTGGTGGRATGCCCTC |  |  |

Degenerated base: B = C or G or T, H = A or C or T, K = T or G, M = A or C, N = A or C or G or T, R = A or G, V = A or C or G, W = A or T, Y = C or T
