# Supplementary material for: Complete genome analysis demonstrates multiple introductions of enterovirus 71 and coxsackievirus A16 recombinant strains into Thailand during the past decade
Source: Emerg Microbes Infect. 2018 Dec 14;7:214. doi: 10.1038/s41426-018-0215-x (PMC6294798; doi:10.1038/s41426-018-0215-x)
Supplement: Supplementary file 3 — Supplementary Table S2 [file 41426_2018_215_MOESM3_ESM.doc]

**Supplementary Table S2.** Panel of amplification and sequencing primers for CA16

| **Fragment**  **no.** | **Primer name** | **Nucleotide position** | **Sequence of primers**  **(5’ --->3’)** | **Product size (bp)** | **Annealing temperature** |
| --- | --- | --- | --- | --- | --- |
| 1 | CA16_A | 1-20 | TTAAAACAGCCTGTGGGTTG | ~1,075 | 54°C |
|  | CA16_A1R | 1057-1074 | CCAYTCCCCATAKGCTAT |  |  |
|  | CA16_A590R | 592-611 | CAATTGTCACCATAAGCAGC |  |  |
| 2 | CA16_B1F | 543-560 | GCGGAACCGACTACTTTG | ~1,140 | 53°C |
|  | CA16_B1R | 1666-1683 | RCACATGGGAGCTATGGT |  |  |
| 3 | CA16_B2F | 1423-1440 | CAGGTTGGTGCAGTHCT | ~1,014 | 49°C |
|  | CA16_B2R | 2417-2435 | GTTTGCTCAATRTCCTCHG |  |  |
| 4 | CA16_B3F | 2182-2220 | CATGTGATCTGGGACTTYG | ~1,006 | 52°C |
|  | CA16_B3R | 3169-3188 | ATGGAGTGTGGTGACTTCTC |  |  |
| 5 | CA16_B4F | 3007-3024 | CCAGCTCAAGTGTCAGTC | ~1,283 | 52°C |
|  | CA16_B4R | 4273-4290 | GGCAGCAGAYTGYTCAAG |  |  |
| 6 | CA16_C1F | 4072-4091 | ATAGTGCAAAAACAGAGCGC | ~1,284 | 52°C |
|  | CA16_C1R | 5338-5356 | GCTTAGGBGCRCCAGAATA |  |  |
| 7 | CA16_C2F | 5191-5211 | CARGGGTGGATAATTCCAGAA | ~1,124 | 54°C |
|  | CA16_C2R | 6296-6315 | CARGGCACTATAGGGGTACC |  |  |
| 8 | CA16_C3F | 6181-6200 | CTCCACTATGCAAAYCAGYT | ~1,248 | 53°C |
|  | CA16_C3R | 7402-7429 | TTTTTTTTTTGCGTATTCTGGT ATAAC |  |  |
|  | CA16_C3F2 | 6796-6814 | CTTGGTGGAATGCCYTCAG |  |  |

Degenerated base: B = C or G or T, H = A or C or T, K = T or G, M = A or C, N = A or C or G or T, R = A or G, V = A or C or G, W = A or T, Y = C or T
