# Supplementary material for: Complete genome analysis demonstrates multiple introductions of enterovirus 71 and coxsackievirus A16 recombinant strains into Thailand during the past decade
Source: Emerg Microbes Infect. 2018 Dec 14;7:214. doi: 10.1038/s41426-018-0215-x (PMC6294798; doi:10.1038/s41426-018-0215-x)
Supplement: Supplementary file 4 — Supplementary Table S3 [file 41426_2018_215_MOESM4_ESM.doc]

**Supplementary Table S3.** *Enterovirus A* (EV-A) prototypes and oldest EV71 genotypes used for phylogenetic construction and recombination analysis

| **Serotype** | **Strain name** | **Country** | **Isolation year** | **Genotype/**  **Subgenotype** | **GenBank accession no.** | **Note** |
| --- | --- | --- | --- | --- | --- | --- |
| EV71 | BrCr-USA-1970 | USA | 1970 | A | U22521 | oldest |
|  | 10857/NED/1996 | The Netherlands | 1966 | B0 | AB575912 | oldest |
|  | 11977/NED/1971 | The Netherlands | 1971 | B1 | AB575913 | oldest |
|  | 20233/NED/1983 | The Netherlands | 1983 | B2 | AB575923 | oldest |
|  | MS/7423/87 | USA | 1987 | B2 | U22522 | Reference |
|  | MY821-3/1997 | Singapore | 1997 | B3 | DQ341367 | oldest |
|  | 5865/sin/000009/SIN/2000 | Singapore | 2000 | B4 | AF316321 | oldest |
|  | 5511-SIN-00 | Singapore | 2000 | B5 | DQ341364 | oldest |
|  | NED/1991 | The Netherlands | 1991 | C1 | AB575935 | oldest |
|  | Tainan/5746/98/TW/1998 | Taiwan | 1998 | C2 | AF304457 | oldest |
|  | Tainan/4643/98 | Taiwan | 1998 | C2 | AF304458 | Reference |
|  | 06-KOR-00/KOR/2000 | South Korea | 2000 | C3 | DQ341355 | oldest |
|  | SHZH98/CHN/1998 | China | 1998 | C4 | AF302996 | oldest |
|  | 2007-07364/TW/2007 | Taiwan | 2007 | C5 | EU527983 | oldest |
| CA2 | Fleetwood/USA/1947 | USA | 1947 | A2 | AY421760 | Prototype |
| CA3 | Olson/USA/1948 | USA | 1948 | A3 | AY421761 | Prototype |
| CA4 | High Point/USA/1948 | USA | 1948 | A4 | AY421762 | Prototype |
| CA5 | Swartz/USA/1950 | USA | 1950 | A5 | AY421763 | Prototype |
| CA6 | Gdula/USA/1949 | USA | 1949 | A6 | AY421764 | Prototype |
| CA7 | Parker/USA/1949 | USA | 1949 | A7 | AY421765 | Prototype |
| CA8 | Donovan/USA/1949 | USA | 1949 | A8 | AY421766 | Prototype |
| CA10 | Kowalik/USA/1959 | USA | 1950 | A10 | AY421767 | Prototype |
| CA12 | Texas-12/USA/1948 | USA | 1948 | A12 | AY421768 | Prototype |
| CA14 | G-14/SOA/1950 | South Africa | 1950 | A14 | AY421769 | Prototype |
| CA16 | G10/SOA/1951 | South Africa | 1951 | A16 | U05876 | Prototype |

Abbreviation: EV, enterovirus; CA, coxsackievirus
