# Supplementary material for: Complete genome analysis demonstrates multiple introductions of enterovirus 71 and coxsackievirus A16 recombinant strains into Thailand during the past decade
Source: Emerg Microbes Infect. 2018 Dec 14;7:214. doi: 10.1038/s41426-018-0215-x (PMC6294798; doi:10.1038/s41426-018-0215-x)
Supplement: Supplementary file 5 — Supplementary Table S4 [file 41426_2018_215_MOESM5_ESM.doc]

**Supplementary Table S4. Percentages of nucleotide sequence identities between enterovirus Thai isolates and prototype strains**

| **Virus** | **Region** | **% Nucleotide identity to prototype strains with genotypes*** | | | | | | | | |
| --- | --- | --- | --- | --- | --- | --- | --- | --- | --- | --- |
|  |  | **EV71-A** | **EV71-B5** | **EV71-C2** | **CA3** | **CA4** | **CA6** | **CA12** | **CA14** | **CA16** |
| EV71-B5 non-recombinant strains  (19 isolates) | 5’-UTR | 84.9-86.4 | 95.8-96.5 | 83.2-85.2 | 83.6-84.6 | 84.6-84.9 | 84.4-85.5 | 81.5-82.9 | 83.8-85.5 | 85.3-86.0 |
|  | P1 | 81.1-81.8 | 93.6-95.0 | 81.6-82.3 | 62.7-63.4 | 62.5-63.2 | 61.9-62.3 | 62.2-62.7 | 65.6-66.2 | 68.0-68.7 |
|  | VP4 | 80.6-84.5 | 88.8-94.6 | 79.7-82.1 | 62.3-65.6 | 64.2-66.1 | 63.2-66.1 | 61.8-65.2 | 62.8-64.7 | 69.0-71.4 |
|  | VP2 | 80.4-81.6 | 93.0-94.7 | 80.4-83.4 | 64.3-66.4 | 66.5-68.0 | 65.4-66.6 | 65.3-66.5 | 69.8-70.9 | 68.6-70.2 |
|  | VP3 | 80.8-81.6 | 93.2-96.1 | 80.8-81.8 | 65.0-66.5 | 65.5-66.1 | 63.3-64.8 | 65.2-66.1 | 64.8-65.7 | 70.3-71.2 |
|  | VP1 | 81.0-82.3 | 94.2-95.6 | 82.4-83.5 | 58.2-59.2 | 55.1-55.9 | 55.4-56.8 | 57.3-58.1 | 62.6-63.7 | 64.1-65.3 |
|  | P2 | 76.9-77.6 | 94.1-95.4 | 78.3-79.0 | 77.1-77.9 | 81.3-82.6 | 77.5-78.6 | 77.5-78.2 | 82.4-83.2 | 81.9-82.6 |
|  | 2A | 75.1-77.3 | 92.8-95.7 | 79.3-80.4 | 76.4-78.0 | 78.6-79.7 | 77.1-79.3 | 74.8-76.6 | 79.1-80.6 | 78.0-80.4 |
|  | 2B | 75.4-77.4 | 91.9-95.9 | 73.0-75.7 | 78.1-79.4 | 80.4-83.8 | 77.1-79.1 | 77.1-78.4 | 81.4-82.8 | 82.4-84.5 |
|  | 2C | 77.0-78.3 | 94.7-96.1 | 78.5-79.6 | 76.8-78.1 | 82.5-83.4 | 77.2-78.6 | 78.3-79.6 | 83.7-84.5 | 83.0-84.1 |
|  | P3 | 78.5-79.7 | 93.2-94.2 | 77.6-77.9 | 79.5-80.6 | 79.2-80.0 | 79.5-80.4 | 79.9-80.6 | 78.6-79.2 | 78.0-78.6 |
|  | 3A | 83.3-84.8 | 93.4-95.7 | 73.2-76.7 | 77.9-81.0 | 85.2-87.5 | 76.3-81.0 | 78.6-81.3 | 83.3-87.2 | 81.7-85.6 |
|  | 3B | 69.6-74.2 | 90.9-96.9 | 77.2-80.3 | 71.2-75.7 | 75.7-83.3 | 74.2-78.7 | 74.2-78.7 | 80.3-83.3 | 77.2-80.3 |
|  | 3C | 75.4-77.0 | 93.8-94.7 | 74.6-77.0 | 77.9-80.3 | 78.5-79.9 | 76.3-79.7 | 77.4-79.0 | 79.9-81.4 | 75.9-77.9 |
|  | 3D | 78.9-80.5 | 92.3-93.9 | 78.7-79.2 | 79.8-81.3 | 78.1-79.2 | 80.3-81.9 | 80.5-81.7 | 76.7-77.3 | 77.4-78.2 |
| EV71-B5 recombinant strain (SiICRC11/TH/2012) | 5’-UTR | 85.5 | 95.3 | 83.6 | 84.7 | 85.4 | 85.0 | 82.1 | 84.4 | 86.0 |
|  | P1 | 81.7 | 94.0 | 81.7 | 63.0 | 62.5 | 62.2 | 62.6 | 65.7 | 68.3 |
|  | VP4 | 84.0 | 90.8 | 83.0 | 63.2 | 64.7 | 65.2 | 63.7 | 63.7 | 69.5 |
|  | VP2 | 81.3 | 94.2 | 80.5 | 65.2 | 67.3 | 66.2 | 66.4 | 69.8 | 70.3 |
|  | VP3 | 81.4 | 94.0 | 81.1 | 65.7 | 65.8 | 64.3 | 66.1 | 65.4 | 70.9 |
|  | VP1 | 81.8 | 94.5 | 83.0 | 58.9 | 54.7 | 56.7 | 57.7 | 63.0 | 64.3 |
|  | P2 | 78.4 | 89.2 | 78.3 | 78.9 | 80.3 | 79.9 | 79.4 | 81.4 | 80.7 |
|  | 2A | 76.2 | 94.8 | 80.4 | 77.3 | 79.5 | 78.2 | 75.1 | 81.1 | 78.6 |
|  | 2B | 76.0 | 93.9 | 75.0 | 78.7 | 82.4 | 78.7 | 78.1 | 80.4 | 82.8 |
|  | 2C | 80.1 | 85.2 | 78.4 | 79.7 | 80.0 | 81.1 | 81.7 | 81.9 | 81.1 |
|  | P3 | 80.6 | 80.2 | 77.4 | 81.2 | 77.5 | 81.4 | 81.8 | 78.0 | 77.2 |
|  | 3A | 80.6 | 78.6 | 75.1 | 82.9 | 77.1 | 80.6 | 84.1 | 76.3 | 76.7 |
|  | 3B | 83.3 | 77.2 | 77.2 | 87.8 | 77.2 | 87.8 | 87.8 | 74.2 | 77.2 |
|  | 3C | 81.2 | 77.0 | 74.1 | 80.6 | 76.1 | 81.0 | 81.2 | 77.4 | 76.5 |
|  | 3D | 80.3 | 81.9 | 79.2 | 80.8 | 78.1 | 81.5 | 81.4 | 78.7 | 77.7 |

Note: ^*^ EV-A prototype strains include EV71 genotype A strain BrCr-USA-1970 (accession no. U22521), EV71 subgenotype B5 strain 5511-SIN-00 (accession no. DQ341364), EV71 subgenotype C2 strain Tainan/5746/98/TW/1998 (accession no. AF304457), CA3 strain Olson/USA/1948 (accession number AY421761), CA4 strain High Point/USA/1948 (accession no. AY421762), CA6 strain Gdula/USA/1949 (accession no. AY421764), CA12 strain Texas-12/USA/1948 (accession number AY421768), CA14 strain G-14/SOA/1950 (accession number AY421769) and CA16 strain G10/SOA/1951 (accession no. U05876). The yellow boxes indicated high similarity of nucleotide sequences to prototype strains in particular region.

**Supplementary Table S4 (continued). Percentages of nucleotide sequence identities between enterovirus Thai isolates and prototype strains**

| **Virus** | **Region** | **% Nucleotide identity to prototype strains with genotypes*** | | | | | | | | |
| --- | --- | --- | --- | --- | --- | --- | --- | --- | --- | --- |
|  |  | **EV71-A** | **EV71-B5** | **EV71-C2** | **CA3** | **CA4** | **CA6** | **CA12** | **CA14** | **CA16** |
| EV71-C4a recombinant strains  (2 isolates) | 5’-UTR | 83.3-83.6 | 85.3-86.0 | 85.5 | 83.8 | 83.6-84.1 | 83.3-83.6 | 80.9-81.3 | 82.6-83.6 | 82.2-82.8 |
|  | P1 | 81.1-81.8 | 81.9-82.7 | 88.8-89.2 | 63.8-63.9 | 63.1-63.2 | 61.6-61.9 | 63.4 | 65.3-65.6 | 67.4-67.8 |
|  | VP4 | 81.6-83.5 | 82.1 | 85.9-86.9 | 61.8-62.3 | 63.2-64.2 | 64.7 | 64.7-65.2 | 65.7-67.6 | 68.5-69.5 |
|  | VP2 | 80.9 | 81.6-81.8 | 89.2-89.8 | 67.0-67.8 | 65.7-66.1 | 65.7-66.6 | 67.8-67.9 | 69.6-70.0 | 68.7-69.5 |
|  | VP3 | 80.1-82.0 | 80.4-82.6 | 88.4-88.7 | 67.3-68.3 | 65.7-65.9 | 63.7-64.4 | 65.7-66.2 | 65.0-65.1 | 70.6-72.1 |
|  | VP1 | 82.0 | 83.5-83.6 | 89.4-89.7 | 57.9-58.0 | 57.2-57.4 | 54.9-55.1 | 58.1-58.5 | 61.7-61.9 | 62.5-63.5 |
|  | P2 | 78.4-78.6 | 81.8 | 79.0-79.3 | 78.7-79.5 | 82.8-83.4 | 79.1-79.5 | 78.4-78.6 | 82.0-82.4 | 81.1-81.2 |
|  | 2A | 79.5-80.0 | 80.2-80.8 | 81.7-84.0 | 77.1-79.1 | 82.6-84.0 | 78.2-78.4 | 77.3-77.7 | 80.4-80.8 | 78.8-80.2 |
|  | 2B | 75.7-76.0 | 80.8-81.1 | 73.7-74.0 | 77.4-77.7 | 80.4-80.8 | 77.1-78.1 | 75.0-77.1 | 80.4-81.8 | 78.7-80.1 |
|  | 2C | 78.7-78.8 | 82.5-82.8 | 78.9-79.3 | 79.8-80.3 | 83.5-83.9 | 79.8-80.7 | 79.5-79.9 | 83.2-83.3 | 82.2-82.6 |
|  | P3 | 77.6-77.7 | 79.7-79.9 | 76.0 | 77.3-77.4 | 83.1-83.3 | 76.9-77.5 | 76.9-77.4 | 83.5-83.7 | 83.0 |
|  | 3A | 81.0-81.3 | 84.8-86.8 | 74.0-75.5 | 77.1-77.9 | 82.9-83.3 | 76.3-77.1 | 76.3 | 81.7-84.4 | 81.7-82.1 |
|  | 3B | 68.1-71.2 | 84.8-89.3 | 74.2-77.2 | 71.2-74.2 | 80.3-87.8 | 74.2-75.7 | 72.7-74.2 | 81.8-86.3 | 75.7-81.8 |
|  | 3C | 75.9-77.2 | 80.6-82.5 | 74.1-75.9 | 78.1-78.8 | 82.3-83.2 | 77.0-77.2 | 77.4-77.5 | 82.6-83.2 | 81.0-82.5 |
|  | 3D | 77.4-78.2 | 77.5-77.7 | 76.1-77.2 | 76.9-77.4 | 83.3-83.4 | 76.9-78.0 | 77.0-77.8 | 83.9-84.1 | 83.9-84.0 |
| EV71-C4b recombinant strains  (2 isolates) | 5’-UTR | 84.2-85.5 | 86.3 | 85.5-86.0 | 84.2-85.3 | 84.4-84.9 | 84.3-85.0 | 82.7-83.2 | 83.0-84.1 | 82.9-83.9 |
|  | P1 | 82.0-82.2 | 82.4 | 89.1-89.6 | 64.1-64.2 | 62.9 | 60.9-61.2 | 63.0-63.2 | 65.6-65.7 | 68.1-68.6 |
|  | VP4 | 82.1-82.6 | 84.0 | 88.4-89.3 | 62.3-63.2 | 63.2-63.7 | 64.7-65.7 | 66.1-67.1 | 64.2-65.7 | 68.5-69.5 |
|  | VP2 | 80.9 | 81.6-82.0 | 88.7-89.5 | 66.7-66.9 | 65.6-65.7 | 64.5-64.9 | 67.1-67.5 | 69.2-70.0 | 70.0-70.2 |
|  | VP3 | 82.6-82.7 | 81.5-81.9 | 89.5-89.8 | 67.2-67.6 | 65.4-65.5 | 63.2-63.7 | 65.4-65.7 | 66.3-66.6 | 72.0-72.5 |
|  | VP1 | 82.4-82.9 | 82.9-83.5 | 89.3-89.6 | 59.1-59.2 | 57.2-57.5 | 55.1-55.3 | 58.0-58.7 | 61.5-61.9 | 63.2-63.9 |
|  | P2 | 78.5-78.7 | 82.0-82.1 | 79.4-79.6 | 78.3-78.6 | 83.5-83.7 | 79.1-79.3 | 78.4-78.7 | 83.2-83.7 | 83.5-83.7 |
|  | 2A | 78.4-78.8 | 80.8-81.3 | 82.8-83.3 | 78.2-79.3 | 83.3-83.7 | 79.5-80.0 | 78.0-78.2 | 81.1-81.5 | 80.8-80.4 |
|  | 2B | 76.0-76.4 | 80.8-81.1 | 73.4 | 77.4-77.7 | 82.4-82.8 | 78.4-79.1 | 76.4-77.1 | 82.1-84.1 | 82.8 |
|  | 2C | 79.1-79.6 | 82.7-82.9 | 79.5-80.0 | 78.5-78.6 | 83.7-84.1 | 79.0-79.3 | 79.2-79.4 | 84.5 | 85.3-85.5 |
|  | P3 | 77.3-77.7 | 79.2-79.5 | 76.8 | 77.9-78.0 | 84.0 | 77.9-78.1 | 78.3-78.7 | 83.7-83.9 | 83.2-83.3 |
|  | 3A | 79.4 | 82.9 | 75.5-75.9 | 79.8 | 82.1-82.9 | 79.0 | 79.8 | 84.8-85.6 | 84.4 |
|  | 3B | 66.6 | 75.7-77.2 | 71.2-74.2 | 66.6 | 83.3-84.8 | 69.6 | 69.6 | 84.8-86.3 | 84.8-86.3 |
|  | 3C | 76.1-77.5 | 77.0-77.5 | 75.4-75.9 | 76.8-77.7 | 83.6-83.9 | 77.7-78.1 | 79.5 | 81.7-83.4 | 80.5-81.0 |
|  | 3D | 77.9-78.0 | 79.3-79.9 | 77.7-77.8 | 78.2-78.7 | 84.3-84.4 | 78.1-78.5 | 78.0-78.5 | 83.9 | 83.8-84.0 |

Note: ^*^ EV-A prototype strains include EV71 genotype A strain BrCr-USA-1970 (accession no. U22521), EV71 subgenotype B5 strain 5511-SIN-00 (accession no. DQ341364), EV71 subgenotype C2 strain Tainan/5746/98/TW/1998 (accession no. AF304457), CA3 strain Olson/USA/1948 (accession number AY421761), CA4 strain High Point/USA/1948 (accession no. AY421762), CA6 strain Gdula/USA/1949 (accession no. AY421764), CA12 strain Texas-12/USA/1948 (accession number AY421768), CA14 strain G-14/SOA/1950 (accession number AY421769) and CA16 strain G10/SOA/1951 (accession no. U05876). The yellow boxes indicated high similarity of nucleotide sequences to prototype strains in particular region.

**Supplementary Table S4 (continued). Percentages of nucleotide sequence identities between enterovirus Thai isolates and prototype strains**

| **Virus** | **Region** | **% Nucleotide identity to prototype strains with genotypes*** | | | | | | | | |
| --- | --- | --- | --- | --- | --- | --- | --- | --- | --- | --- |
|  |  | **EV71-A** | **EV71-B5** | **EV71-C2** | **CA3** | **CA4** | **CA6** | **CA12** | **CA14** | **CA16** |
| EV71-C2 | 5’-UTR | 82.7 | 85.0 | 95.3 | 82.4 | 83.5 | 82.7 | 82.4 | 84.7 | 82.6 |
|  | P1 | 81.7 | 82.2 | 93.5 | 64.2 | 63.5 | 62.3 | 63.4 | 64.9 | 67.7 |
|  | VP4 | 82.6 | 82.1 | 90.8 | 64.2 | 64.2 | 64.7 | 67.1 | 62.8 | 66.1 |
|  | VP2 | 81.3 | 83.3 | 94.7 | 67.4 | 66.9 | 66.6 | 67.4 | 68.6 | 69.4 |
|  | VP3 | 81.8 | 82.2 | 93.3 | 67.6 | 66.2 | 64.1 | 66.8 | 66.1 | 71.4 |
|  | VP1 | 81.8 | 81.2 | 93.1 | 58.6 | 57.1 | 56.5 | 57.6 | 61.2 | 63.7 |
|  | P2 | 78.3 | 79.1 | 92.6 | 78.2 | 78.2 | 80.0 | 78.4 | 78.7 | 78.6 |
|  | 2A | 78.4 | 80.2 | 90.8 | 78.0 | 79.5 | 81.1 | 78.8 | 82.0 | 78.0 |
|  | 2B | 76.7 | 75.4 | 93.2 | 77.4 | 74.7 | 77.7 | 78.1 | 75.7 | 78.1 |
|  | 2C | 78.8 | 79.7 | 93.2 | 78.6 | 78.7 | 80.2 | 78.4 | 78.2 | 79.0 |
|  | P3 | 76.7 | 78.0 | 92.6 | 78.7 | 77.2 | 78.2 | 78.3 | 77.6 | 77.7 |
|  | 3A | 76.3 | 72.4 | 92.6 | 79.0 | 75.5 | 78.2 | 75.9 | 74.8 | 75.9 |
|  | 3B | 66.6 | 83.3 | 96.9 | 72.7 | 80.3 | 77.2 | 78.7 | 86.3 | 83.3 |
|  | 3C | 74.1 | 75.9 | 92.7 | 75.2 | 76.5 | 76.3 | 77.9 | 76.3 | 74.1 |
|  | 3D | 78.3 | 79.6 | 92.4 | 80.4 | 77.7 | 79.0 | 78.9 | 78.2 | 79.2 |
| CA16  recombinant strains  (7 isolates) | 5’-UTR | 83.4-84.8 | 81.9-84.0 | 81.2-82.2 | 84.2-85.8 | 84.5-87.0 | 84.2-86.2 | 84.4-85.8 | 83.6-85.8 | 84.1-86.0 |
|  | P1 | 68.8-69.4 | 68.6-69.1 | 68.1-68.3 | 61.9-62.3 | 62.2-63.1 | 62.6-63.0 | 64.4-65.7 | 65.2-66.4 | 76.8-77.9 |
|  | VP4 | 69.5-70.5 | 70.5-72.4 | 65.2-67.6 | 62.8-65.7 | 64.7-67.1 | 62.8-65.2 | 61.8-64.7 | 59.4-62.8 | 80.6-82.6 |
|  | VP2 | 70.9-71.7 | 70.2-71.3 | 71.3-72.1 | 64.9-65.8 | 65.3-66.4 | 66.7-67.9 | 68.0-70.1 | 69.6-70.8 | 75.8-78.0 |
|  | VP3 | 70.7-72.0 | 70.6-71.3 | 69.8-70.7 | 63.7-65.4 | 64.0-64.8 | 63.4-65.2 | 66.8-67.6 | 68.5-69.5 | 77.4-79.3 |
|  | VP1 | 64.9-65.8 | 63.7-65.3 | 63.8-64.5 | 56.7-57.1 | 56.9-57.8 | 56.8-57.9 | 58.6-60.0 | 60.0-61.0 | 75.4-76.3 |
|  | P2 | 82.6-83.5 | 78.4-79.9 | 77.7-78.8 | 82.0-82.9 | 77.1-78.3 | 81.9-82.7 | 82.0-83.3 | 78.7-79.0 | 79.6-80.1 |
|  | 2A | 78.2-80.6 | 76.4-79.5 | 77.5-78.2 | 79.5-81.7 | 76.2-78.0 | 77.5-79.5 | 78.0-80.2 | 78.6-79.3 | 80.0-81.1 |
|  | 2B | 82.4-83.8 | 76.0-77.4 | 75.0-77.1 | 80.1-83.1 | 76.0-78.1 | 83.5-84.8 | 81.1-82.8 | 77.1-78.1 | 75.7-77.4 |
|  | 2C | 84.2-85.4 | 79.7-80.1 | 78.4-80.1 | 83.1-84.0 | 77.9-79.0 | 82.9-83.5 | 83.2-85.2 | 79.1-79.8 | 79.8-80.8 |
|  | P3 | 82.9-83.5 | 79.4-79.9 | 77.7-78.3 | 83.3-83.8 | 77.2-78.0 | 82.6-83.5 | 82.3-83.5 | 76.8-77.4 | 77.1-78.2 |
|  | 3A | 83.3-84.8 | 78.2-80.6 | 75.5-77.5 | 83.3-84.8 | 78.6-80.2 | 82.1-83.7 | 81.7-85.6 | 78.2-81.0 | 79.4-81.3 |
|  | 3B | 78.7-87.8 | 68.1-74.2 | 69.6-74.2 | 83.3-86.3 | 74.2-81.8 | 80.3-87.8 | 77.2-84.8 | 71.2-78.7 | 71.2-78.7 |
|  | 3C | 83.2-85.0 | 76.6-77.9 | 75.9-76.8 | 83.0-84.3 | 77.0-76.3 | 81.7-83.6 | 82.3-84.1 | 76.1-77.2 | 75.5-76.5 |
|  | 3D | 82.3-83.5 | 80.5-81.3 | 78.7-79.5 | 82.6-83.6 | 76.8-77.9 | 82.4-84.0 | 81.5-83.3 | 76.7-77.4 | 77.4-78.3 |

Note: ^*^ EV-A prototype strains include EV71 genotype A strain BrCr-USA-1970 (accession no. U22521), EV71 subgenotype B5 strain 5511-SIN-00 (accession no. DQ341364), EV71 subgenotype C2 strain Tainan/5746/98/TW/1998 (accession no. AF304457), CA3 strain Olson/USA/1948 (accession number AY421761), CA4 strain High Point/USA/1948 (accession no. AY421762), CA6 strain Gdula/USA/1949 (accession no. AY421764), CA12 strain Texas-12/USA/1948 (accession number AY421768), CA14 strain G-14/SOA/1950 (accession number AY421769) and CA16 strain G10/SOA/1951 (accession no. U05876). The yellow boxes indicated high similarity of nucleotide sequences to prototype strains in particular region.
